# General Pathology of Acute Bacillary Gangrene Arising in Gunshot Injuries of Muscle

**Published:** 1917-12

**Authors:** 


***General Pathology of Acute Bacillary Gangrene Arising in Gunshot Injuries of Muscle.*** By Capt. E. F. Bashford, R. A. M. C. Abs. from the *British Journal of Surgery*, April, 1917.

B. brings to the study of gaseous gangrene, which he prefers to call " 'anaerobic' or better 'acute bacillary gangrene' or 'war gangrene' ", the results of an extended histological study of the process of healing in muscles.

He finds that the immediate injury to the muscle is sharply circumscribed. " Around the missile track is produced a zone of disintegrated muscle, a freshly made 'beef emulsion', which is sharply marked off from the adjacent living fibers, though the interspaces between the bundles and fibers open into this ideal culture medium ".

Examination of a portion of segmental muscle from a typical case of gaseous gangrene in the rectus abdominis is described as consisting of three segments : (1) injured, (2) reacting, (3) normal. In the injured section there appeared an extraordinary variety of organisms, among them some resembling B. perfringens (aerogenes capsulatus) and B. œdematis maligni, but B. thinks these may be Hibler's bacillus or " any of many others ". " What is important to record ", he believes, " is that in sections this great variety is at its maximum among the dead muscle fibres, not *in* them.... The bacilli are not essentially 'muscle feeders' as the phrase goes, but live in the lymph spaces which represent the endomysium, and in the 'beef emulsion' ".

The author notes that the infection is stayed effectively where the circulation is maintained unimpaired; " but " he adds, " the influence of the bacterial products has extended into this zone, and has there interfered with the regenerative process in the muscle. "

In the case of long muscle the same processes are observed, but " the various stages of tissue injury and bacterial invasion may pass rapidly from one to another throughout a great length of tissue; the bacterial invasion, acquiring increased magnitude with every step, comes to involve not only single muscles, but groups and even the entire limb.

" After infection has been fully established in a long muscle, the intensity of the processes resulting therefrom can often be well followed from the central fibers towards the sheath, which frequently remains uninfected, but may be the chief seat of the spread of the bacilli. The septa between muscles, the connective tissue surrounding veins, or such structures as Hunter's canal, may be the paths of extension ".

With regard to the gas bubbles, the author states : " In the muscle they are invariably between bundles of fibres, never *in* them; the arrangement of leucocytes and bacteria around the margin of a bubble is always the same—they have been pushed aside during life; as have also the muscle fibers, which, however, nowhere show signs of having been subject to pressure, signs of which are also absent in the exudate and the capillaries between the neighboring fibers ".

As in the case of the segmental muscles the author finds that there is no evidence that the bacilli are essentially 'muscle feeders'; indeed, they are present in the greatest numbers, not where the muscle has been immediately killed by the missile, but in the interfibrillary spaces of the portion intermediate between this zone and the insertion—i. e., they have extended in the lymphatic spaces.

" The bacilli spread along the track of the larger vessels. Once they get into a new longitudinal space, they spread along the capillaries, being closely applied to the sarcolemma of adjacent fibrils or applied to the walls of capillaries, and often arranged longitudinally, but nowhere are they found in the capillaries. This description, it must be recalled, is of a muscle cut off from its blood-supply. "

Examination of portions of muscle retaining a blood supply, especially along the margin of bacillary invasion, leads the author to note that the absence of leucocytosis is a striking feature at the advancing margin of bacillary invasion and in the edema, and he believes that the leucocytes can not set up an effective protective barrier against the invasion of healthy tissue by massive doses of these bacilli.

The author concludes as follows : "There is histological evidence that the products of the bacilli damage the vessels and the muscle fibers and the endomysium ahead of their massive advance, and less evidence that the lymphatics are similarly involved. The process of bacillary invasion is further assisted by the motility of some of them in the fluid, poured out into, but not absorbed from, the tissues, and by the disorganization of the vascular arrangements.... In places the end result is nothing short of extensive intra-muscular hemorrhage, with the further provision of fresh masses of tissue ideally suited for bacterial growth. At times this process is augmented by rapid and by remote thrombosis. "

The author cites several cases in which marked thrombosis has attended cases of gaseous gangrene, appearing in the proximal veins, and capillaries and aiding, as he believes, in the extension of the bacillary invasion.

" The chief importance of thrombosis lies in the provision of fresh areas of dead tissues suitable for the extension of bacterial invasion, and in the essential stage it provides in an ever-widening and vicious circle; and it is obvious from the examples cited that thrombosis may occur both extensively and suddenly. "

In his summary, the author gives the following theoretical conception of the spread of the disease : "The combinations in which 'anaerobic' bacilli occur in gunshot injuries are exceedingly virulent. While the bacilli themselves multiply mainly in the areolar tissue of the endomysium, their products actively destroy the endothelium of vessels, muscle fibers, and blood. Destruction of capillaries, veins, and lymphatics is the outstanding feature of the rapid spread of the infection which is also accompanied by swelling and degeneration of muscle fibers, and later by the formation of gas. Constitutional symptoms arising from interference with the cardiovascular and heat-regulating mechanism ultimately supervene and usher in the end ". He believes that this theory helps to reconcile the contradictions noted by Wallace between the D'Este Emery and Taylor theories, while it agrees with Wallace's clinical account. The author however, adds ; " the production of gas is a late, and really a subsidiary, phenomenon, which attracts attention from its mere peculiarity. I have not found it plays any part in the advance of the infection, although it contributes to the later swollen condition. It arises in tissue long dead ; for this reason the term 'gas gangrene' is unfortunate, owing to its implying the necessity of awaiting the detection of gas before making a diagnosis ".

